# Ocular modifications in a young girl with cryopyrin-associated periodic syndromes responding to interleukin-1 receptor antagonist anakinra

**DOI:** 10.1007/s12348-010-0018-2

**Published:** 2011-04-09

**Authors:** Celine Terrada, Benedicte Neven, Nathalie Boddaert, Eric H Souied, Anne Marie Prieur, Pierre Quartier, Phuc Lehoang, Bahram Bodaghi

**Affiliations:** 1 Department of Ophthalmology, APHP, Pitie-Salpetriere Hospital, 75013 Paris, France; 2 Department of Ophthalmology, Creteil University Eye Clinic, Creteil, France; 3 Pediatric Immuno-hematology and Rheumatology Units, APHP, Necker Hospital, 75015 Paris, France; 4 INSERM U768, Université Paris Descartes, Paris, France; 5 Department of Genetic and Pediatric Radiology, APHP, Necker Hospital, 75015 Paris, France; 6 Department of Ophthalmology, APHP, Pitie salpetrier Hospital, Boulevard de l’hopital, 75013 Paris, France

## Abstract

An 8-year-old patient with genetically confirmed chronic infantile neurological cutaneous and articular syndrome was treated with interleukin-1 receptor antagonist, anakinra. She initially presented with recurrent episodes of fever, rash, chronic fatigue, frequent headaches, ocular involvement (corneal infiltrate and papillary edema), and permanent increased biologic inflammatory markers. Following treatment with anakinra, all symptoms and inflammation resolved. Ophthalmologic signs normalized. This ophthalmologic description (optic nerve and cornea) has never been illustrated, even if ocular affections are classic in the cryopyrin-associated periodic syndromes.

## Introduction

Cryopyrin-associated periodic syndromes (CAPS) have been proposed to describe cryopyrin-related diseases with heterogenous phenotype severity. The mildest condition is the familial cold auto-inflammatory syndrome, patients with Muckle–Wells syndrome have an intermediate phenotype, the chronic infantile neurological cutaneous and articular syndrome (CINCA) also known as neonatal onset multisystem inflammatory disease (NOMID) is the most severe disease in this spectrum [1, 2]. The patients are presenting urticaria and life-like skin rash from early in life, variable articular involvement (arthralgia/arthritis is associated in 30% of cases with hypertrophic arthropathies), chronic aseptic meningitis, and neurosensorial involvement associated with biologic markers of neutrophil-driven inflammation. Broad ophthalmologic abnormalities have been described in CINCA/NOMID. An international collaborative study based on a questionnaire including 31 patients described the optic disc changes as the most common feature (83%), including optic disc edema, pseudopapilledema, and optic atrophy. Anterior segment manifestations varying from mild to severe (42%); chronic anterior uveitis (55%) [3]. CAPS are all caused by dominantly inherited or de novo mutations in *CIAS1*, a gene that encode for NLRP3 (also known as cryopyrin/NALP3/PYPAF1) [4, 5]. NLRP3 is a component of interleukin-1 (IL-1) inflammasome that regulates IL-1β production, a strong pro-inflammatory cytokine. Mutations of CIAS1 associated with CAPS result in a gain-of-function effect. Thanks to substantial advances in understanding genetic basis and mechanisms of these disorders, new therapeutics targeting the IL-1 pathway could be proposed as recombinant nonglycosylated homolog of human interleukin-1 receptor antagonist (IL-1Ra), anakinra that competitively inhibits binding of IL-1α and IL-1β to IL-1 receptor. Previous studies showed remarkable efficacy of anakinra in patients suffering from CIAS1-associated diseases [6].

In the presenting case of CINCA/NOMID successfully treated by anakinra, we report and document normalization of ophthalmologic involvement undertreatment.

### Case report

**Fig. 1:**
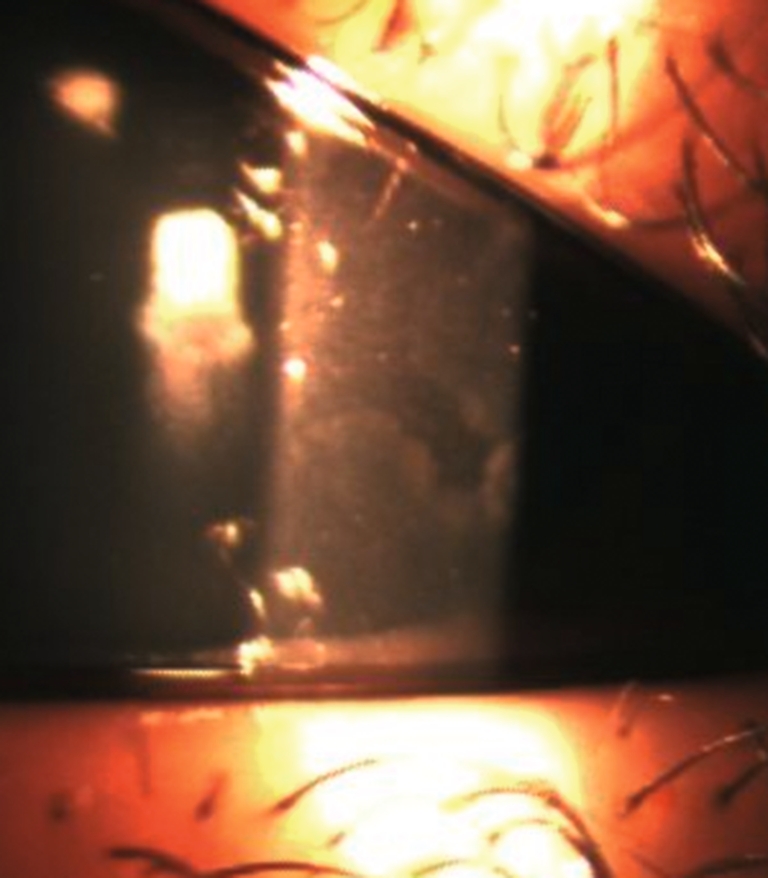
Baseline biomicroscopic examination. Anterior segment photograph illustrates anterior stromal infiltrations of the cornea, these were well defined and corneal epithelium is respected (fluorescein test was negative). The anterior chamber was calm (absence of tyndall and synechiae)

The patient, an 8-year-old Caucasian girl, was referred to the Pediatric Immuno-hematology and Rheumatology Department for diagnosis and treatment of CINCA/NOMID syndrome. Diagnosis was suspected based on medical history of the child. She was born preterm at 30.5 weeks of gestational age with normal weight and height for age (2,010 g and 41.5 cm). She was issued from a twin pregnancy. Because the twin child was diagnosed with trisomy 13, in utero reduction was performed. Prematurity might be related to this procedure rather than to the disease. She presented with daily urticaria-like rash associated with fever since 1 week of age. Arthralgia and transient haunch swelling revealed with nonsteroidal anti-inflammatory drugs were reported at the age of 3 years. Since the age of two, she also complained of recurrent episodes of headaches. Diagnosis of CAPS was genetically confirmed (after the parents provided written informed consent according to the recommendations of the Declaration of Helsinki) and revealed a previously reported mutation, *D303N,* in the *CIAS1* gene. Extensive baseline evaluation was performed before starting treatment with IL1-Ra, anakinra. Clinical examination confirmed the rash. Height and weight were 1.3 m and 24.8 kg, respectively. Biologic inflammatory markers were increased [C-reactive protein 40 mg/dl (normal <5)], white blood cell count 11,800 cells/mm^3^, and neutrophil count 7,860 cells/mm^3^). Bone X-Ray confirmed the absence of arthropathy. Lumbar puncture was performed and cerebrospinal fluid (CSF) examination revealed pleocytosis (25 cells/μl with 90% neutrophils), increased proteinorachia (protein level 0.71 mg/l), and high open pressure (21 H20 cm). Brain MRI (with FLAIR imaging and contrast injection) was normal (absence of abnormalities of small vessels of the basal ganglia and periventricular white matter lesions). Cognitive performances were normal. Audiographic examination showed mild bilateral sensorineural deafness (−20 dB). Vision was preserved in both eyes (20/25 Snellen visual acuity). The Goldmann visual field illustrated a mild blind spot enlargement. Slit lamp biomicroscopy revealed bilateral anterior, nummular, stromal keratitis, and absence of anterior uveitis (Fig. 1). On fundus examination and photographs of both eyes, we observed a bilateral papilledema without vitritis. Diagnosis of CINCA/NOMID was performed.

**Fig. 2:**
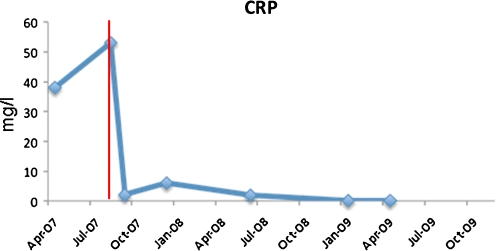
Change in level of C-reactive protein (CRP) with anakinra treatment after 30 months of follow-up. *Vertical discontinued line* indicated the introduction time of anakinra. CRP rapidly decreases 1 month after initiation of the IL-1Ra treatment. CRP was normalized and stabilized during the follow-up

The patient did not receive any steroids or immunosuppressive treatment before initiation of anakinra treatment. Follow-up period is 30 months; initial dose of 2 mg/kg/day in subcutaneous injection has been maintained. All symptoms related to the diseases (rash, headaches, arthralgia, and chronic fatigue) ceased durably in few days. Biologic inflammatory markers [C-reactive protein (CRP), erythrocyte sedimentation rate (ESR)], white blood cells, and neutrophil numerations rapidly decrease 1 month after initiation of the anti-IL-1 treatment. Inflammation markers rates were normalized and stabilized during the follow-up (Fig. 2). The velocity of growth was restarted and the height and weight increased progressively during the treatment. Audiogram remained stable overtime.

**Fig. 3:**
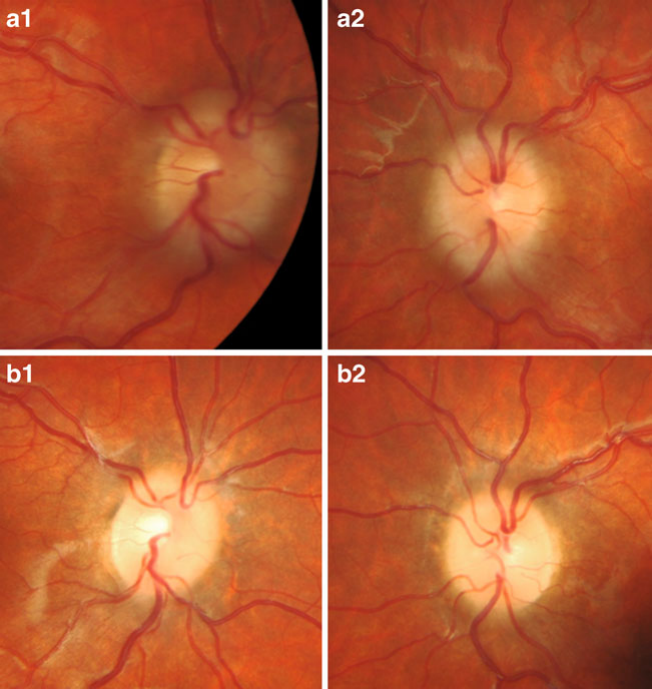
Funduscopy follow-up after anakinra introduction. **a** Baseline ophthalmologic examination: color fundus photograph shows bilateral papilledema without vitritis or vasculitis in the right eye (*A1*) and in the left eye (*A2*). **b** Twelve months follow-up ophthalmologic examination after anakinra has been introduced. Modifications of the cornea were resolute *ad integrum* (not shown). Funduscopy show bilateral pale optic disc without edema, (*B1*) right eye and (*B2*) left eye

Six months after the introduction of anakinra, the corneal infiltrates disappeared. On fundus examination and photographs of both eyes, we observed a pale optic disc corresponding to the resolution of the papilledema (Fig. 3). The campimetry was not modified by the treatment and the blind spot enlargement was stable between the different exams. No adverse events and severe infection occurred. Treatment was well tolerated.

## Discussion

Here we report a case of CINCA/NOMID with chronic inflammation and neurosensorial involvement but without hypertrophic arthropathy successfully treated with IL-1Ra, anakinra. The recombinant form of the naturally occurring IL-1Ra is called anakinra (rmetHuIL-1Ra), and differs from the native human protein that is not glycosylated and has an additional N-terminal methionine [7]. Anakinra competitively inhibits binding of large number of IL-1 receptors, as these receptors are expressed on all cells except red blood cells. IL-1 is a major inflammatory mediator and induces fever, anorexia, hypotension, leucopenia, and thrombocytopenia. IL-1 stimulates production of IL-6, fibrinogen, and complement components. IL-1 also stimulates the hypothalamic–pituitary–adrenal axis [8] and promotes Th17 differentiation. Th17 is involved in autoimmunity and Th17 cells are pivotal in autoimmune uveitis [9]. Eye involvement in our patient was characterized by papillary edema and cornea infiltrate. Ophthalmologic involvement in CAPS are pleiotropic as episclera, anterior chamber, vitreous, and optic disc can be affected [10, 11]. To the best of our knowledge, corneal infiltrates were not previously reported. The explanation proposed regarding reversal nummular infiltrates included [1] cellular inflammatory infiltration in the stroma or [2] reversal amyloid deposits. The first hypothesis seems to be more rational because in this case, systemic amyloidosis was not present. Papilledema is related to chronic intracranial hypertension due to chronic CSF inflammation. Anakinra was dramatically efficient to treat systemic inflammation, articular pain and recurrent headaches were also relieved. Ophthalmologic improvement was also remarkable as shown in Fig. 3: papilledema resolved in 6 months as corneal infiltrates. Unfortunately, visual field does not improve because of papilledema duration before anti-IL-1Ra treatment and alteration of the optic nerve fibers.

The auto fluorescence fundus photographs did not show hyper signal resulting in auto fluorescent material accumulation (not shown). Unfortunately, visual field does not improve because of papilledema duration and alteration of the optic nerve fibers.

We confirmed improvement in fundus examination and regulation of CSF white cell counts with IL-1 receptor antagonism in the treatment of auto-inflammatory conditions. Also, we suggest, early in the course of the disease, the use of IL-1 receptor antagonist as a therapeutic option to chronic papilledema in order to prevent irreversible consequence of chronic excessive intracranial pressure and final optic atrophy.
